# Metabolic Responses and Pathway Changes of Vero Cells under High-Vitamin B Medium

**DOI:** 10.3390/vaccines10111787

**Published:** 2022-10-25

**Authors:** Shouzhi Yu, Junyu Yan, Zhaona Yang, Yuxiu Zhao, Hui Wang, Xiaoming Yang

**Affiliations:** 1 Beijing Institute of Biological Products Company Limited, Beijing 100176, China; 2 China National Biotec Group Company Limited, Beijing 100024, China

**Keywords:** Vero cell, vitamin B, culture medium, multi-omics analysis

## Abstract

The production efficiency of a cell substrate directly affects the yield of target products such as viruses, while its density is mainly regulated by the type of culture medium and culture conditions. In this study, Vero cells were used as model cells for systematic medium screening, and a high-efficiency medium for biological drug production was identified. Through the results of cell proliferation by a cell counting kit (CCK)-8 assay, 5-Ethynyl-2′-deoxyuridine(EdU) assay, real-time quantitative PCR (RT-qPCR) and Western blotting, we found that adding an appropriate amount of vitamin B to the conventional basic medium can significantly improve and maintain the high-density growth of Vero cells. In addition, the molecular mechanism of the high-density culture of Vero cells promoted by B vitamins is explained for the first time by using the systems multi-omics analysis methods. Here, we determined that B vitamins regulate cell proliferation through the synthesis and metabolism of unsaturated fatty acids, affecting the productivity of cell substrate in industrial production. This study provides an important tool for the screening of key components of cell-based high-efficiency medium.

## 1. Introduction

Vero cells are the ideal substrate for the production of human vaccines recommended by the World Health Organization, and their wide susceptibility promotes the development of vaccine production with Vero cells [1,2,3]. The production types of Vero cells include live attenuated vaccines and inactivated whole-virus vaccines, commonly used for Japanese encephalitis, rabies, polio and EV71 (enterovirus 71) virus culture [3,4,5]. The density growth of Vero cell culture directly affects the production of target products. It has previously been shown that the density of Vero cells significantly affects the titer of poliovirus, COVID-19 and rabies virus, and subsequently affects the production of vaccines [6,7]. The density of Vero cells is closely related to the culture medium and culture conditions. For example, when Vero cells are used to culture the rabies virus, selecting an ipt-af culture medium and using perfusion culture can significantly improve the virus titer [8], and dissolved oxygen and nutrient supply affect the yield by affecting cell growth and metabolite changes [9].

The selection of a high-efficiency exclusive medium is the key to achieve high-efficiency cell culture. Studies have shown that vitamins can affect cell proliferation by regulating cell metabolism. B vitamins affect cell proliferation by affecting cell metabolism and DNA synthesis, especially vitamins B1, B2 and B6. Vitamin B1 (vitamin B1, VB1 and thiamine) can maintain normal glucose metabolism, vitamin B2 (vitamin B2, VB2 and riboflavin) promotes cell regeneration and regulates cell metabolism and vitamin B6 (vitamin B6 and VB6) can participate in the metabolism of tryptophan and regulate cell proliferation [10,11,12]. However, it is not clear whether the combined addition of multiple B vitamins can promote cell proliferation synergistically, and the mechanism of B vitamins promoting cell proliferation has not been fully elucidated. At present, researchers use various means to study cell growth and cellular phenotype, such as metabolomics for the impact of newborn bovine serum on human mesenchymal stromal cells [13] and identification of glycosylated marker proteins of epithelial polarity in MDCK cells by proteomic analysis [14], which provides novel insight into cell substrate research for production. 

In this study, CCK-8, EdU, RT-qPCR and Western blotting methods were used to prove that the combined addition of B vitamins can promote the density culture of Vero cells for the first time. To clarify the molecular mechanism, the systematic biological analysis of proteomics, metabolomics and transcriptomics were used to further determine that B vitamins regulate cell proliferation through the synthesis and metabolism of unsaturated fatty acids, which affect the density of Vero cell culture and ultimately affect the culture efficiency and yield of virus. This study revealed the mechanism of efficient medium supplemented with B vitamins to improve the efficiency cell culture and provided an analytical method for in-depth evaluation of cell culture technology.

## 2. Results

### 2.1. Effect of B Vitamins on Vero Cell Proliferation

The conventional medium for Vero cells is commercialized M199. To determine whether the current addition of vitamin B1, vitamin B2 and vitamin B6 can synergistically promote the proliferation of Vero cells, we added the concentration of 1 to 10 times vitamins in commercialized M199, detecting the Vero cell density after 96 h under the same conditions.

**Figure 1:**
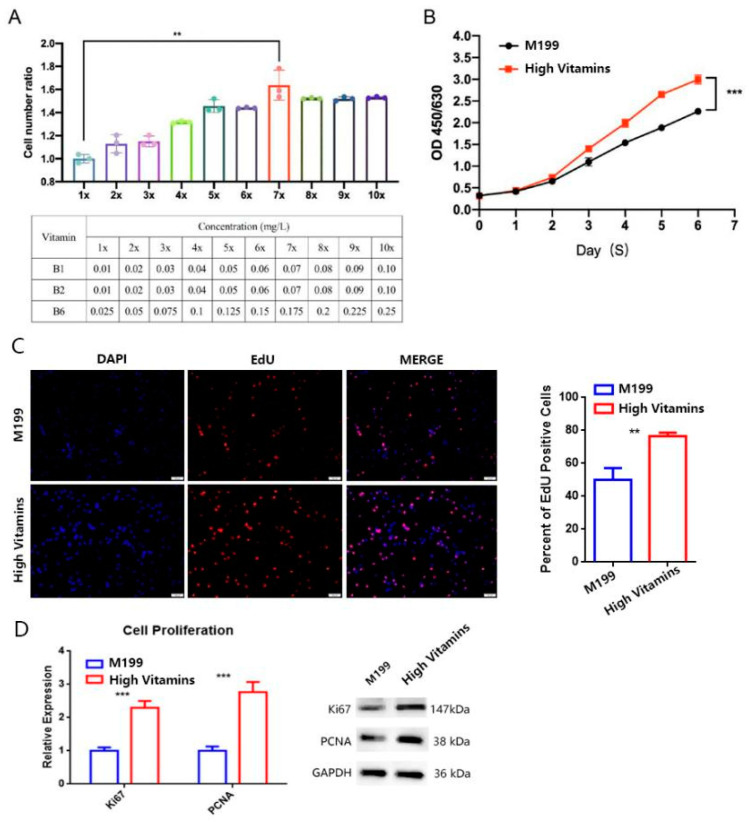
Vitamins supplements promote Vero cell proliferation. (**A**) Effect of different vitamin concentrations on Vero cell proliferation. Different concentrations of vitamin B were added to Vero with the same density. After 96 h of culture under the same conditions, the total number of cells was detected by CountStar. The cell number ratios were calculated and the histogram was drawn for *t*-test. (**B**) Cell proliferation curve detection. Using CCK8 reagent to detect the OD450/650 of Vero cells at the designated time point and draw the proliferation curve, *t*-tests were counted on day 6. (**C**) Effect of cell proliferation by EdU test. The cells were stained with EdU (red) and DAPI (blue). The percentage of Edu-positive cells is shown on the right and the histogram was drawn for the *t*-test. (**D**) Vitamins supplements improve the expression of PCNA and Ki67 both of mRNA (**left**) and protein (**right**) level expression. Housekeeping gene GAPDH is an internal reference. Error bars represent SEM. ** *p* < 0.01; *** *p* < 0.001.

The results showed that the addition of vitamins could promote the high-density growth of Vero cells by improving the concentration to seven doses and the proliferation ratio is dose-dependent (Figure 1A, *p* = 0.0012 **). However, after reaching seven doses of vitamins, the proliferation ability of cells will not be further improved, indicating that the consumption of vitamins by cells has reached saturation. Therefore, the formula of a high-vitamin group is M199 with vitamin B1 (0.07 mg/L), B2 (0.07 mg/L) and B6 (0.175 mg/L) added.

The cell proliferation curve under different culture medium conditions was further verified. The results showed that with the extension of culture time, the proliferation of cells of high-vitamin group (vitamin B1 of 0.07 mg/L, B2 of 0.07 mg/L, B6 of 0.175 mg/L) showed a significant increase (Figure 1B, *p* = 0.0005 ***). By 5-Ethynyl-2′- deoxyuridine(EdU) assay, the EdU positive cell rate of high vitamin group was significantly increased (Figure 1C, *p* = 0.0014 **). Through the comparison of protein level and mRNA level, it was confirmed that the cell proliferation marker proteins PCNA and Ki67 expression was up-regulated (Figure 1D, mRNA level on the left and protein level on the right). The above data show that the addition of multiple vitamin B can promote the proliferation of Vero cells.

### 2.2. Transcriptomics Revealed Vitamin B Affected Metabolic Pathways in Vero Cells

**Figure 2:**
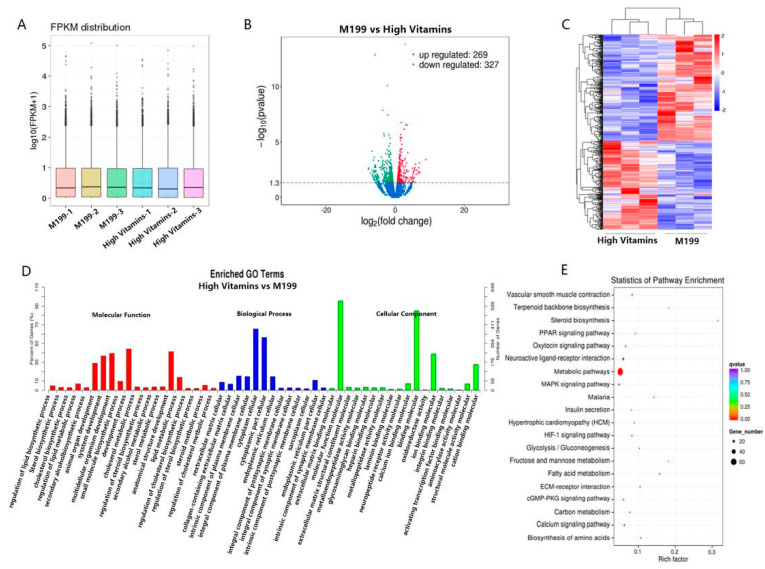
Transcriptomic analysis. (**A**) Box plot. Box plots of expression levels of different samples, where the abscissa is the sample name, and the ordinate is log10 (FPKM + 1). (**B**) The abscissa represents the fold change (log2 Fold Change) of gene expression between different samples or comparison combinations, the larger the absolute value of the abscissa, the greater the fold change of expression between the two comparison combinations; the ordinate represents the significance level of the expression difference. Up-regulated genes are indicated by red dots, down-regulated genes are indicated by green dots and blue dots are genes that did not change significantly. (**C**) The abscissa is the sample, the ordinate is the differential gene, the left side is the clustering of genes according to the similarity of expression, the upper is the clustering of each sample according to the similarity of the expression profile, the expression level is gradually increased from blue to red and the numbers are uniform the relative expression level after transformation. (**D**) The abscissa represents the name of GO items, which are divided into three categories by boxes, BP biological process, CC cell components and MF molecular functions, which are distinguished by different bars and boxes and the ordinate is the number of genes enriched by GO items. (**E**) The vertical axis in the figure represents different pathways, and the horizontal axis represents the proportion of significantly differentially expressed genes in the corresponding pathway to all genes in the pathway. The size of the circle represents the number of genes enriched in the corresponding pathway, and the larger the circle, the more genes are enriched in the pathway. The color represents the significance of enrichment, and the closer to red, the more significant it is.

To further confirm the mechanism of action of vitamin B, we conducted transcriptomic studies on three replicate Vero cell samples in the high vitamin medium group and the normal medium group. First, the expression values of all genes or transcripts in each sample were calculated, and the expression levels of transcripts in different samples were displayed by box plots (Figure 2A), indicating that the transcript samples were consistent.

After the quantitative analysis was completed, the expression matrix of all samples was obtained, and we analyzed the differential genes. Among them, compared with the normal medium group, the high vitamin medium group had a total of 269 genes, and 327 genes were down-regulated, which were displayed by the volcano plot (Figure 2B). We clustered differential genes with similar expression patterns, and the clustering results were represented by a heat map, as shown in Figure 2C.

In an organism, different genes coordinate with each other to perform their biological functions. After classifying the GO terms enriched by differential genes, a histogram of GO enrichment was drawn (Figure 2D). GO can be divided into three parts: molecular function, biological process and cellular component. The most important biochemical metabolic pathways and signal transduction pathways involved in differentially expressed genes can be explored through significant pathway enrichment. We selected the top 20 enriched pathway entries and displayed them in this figure (Figure 2E). Differential signaling pathways are mainly focused on metabolic pathways, neuroactive ligand-receptor interaction and metabolic pathways.

### 2.3. Proteomics Revealed that the Addition of Vitamin B Promoted the Proliferation of Vero Cells by Affecting Cell Metabolism

To explore the mechanism of adding vitamins (vitamin B1, vitamin B2, vitamin B6, vitamin B12 and seven-fold concentration) to promote the proliferation of Vero cells, we tested the proteomics of cell samples cultured in high-vitamin media and M199 to confirm the changes of protein expression levels in cells. To evaluate the significance of the protein expression difference between the two groups of samples, we performed a *t*-test on the relative quantitative value of the total protein in the samples and calculated the corresponding *p* value, which is taken as the significance index, with the default *p* value ≤ 0.05. The number of up- and down-regulated proteins were screened. The up-regulated proteins were selected with FC ≥ 1.5, and the down-regulated proteins were selected with FC ≤ 0.67. The results showed that a high-vitamins group resulted in 394 up-regulation genes and 281 down-regulation genes.

**Figure 3:**
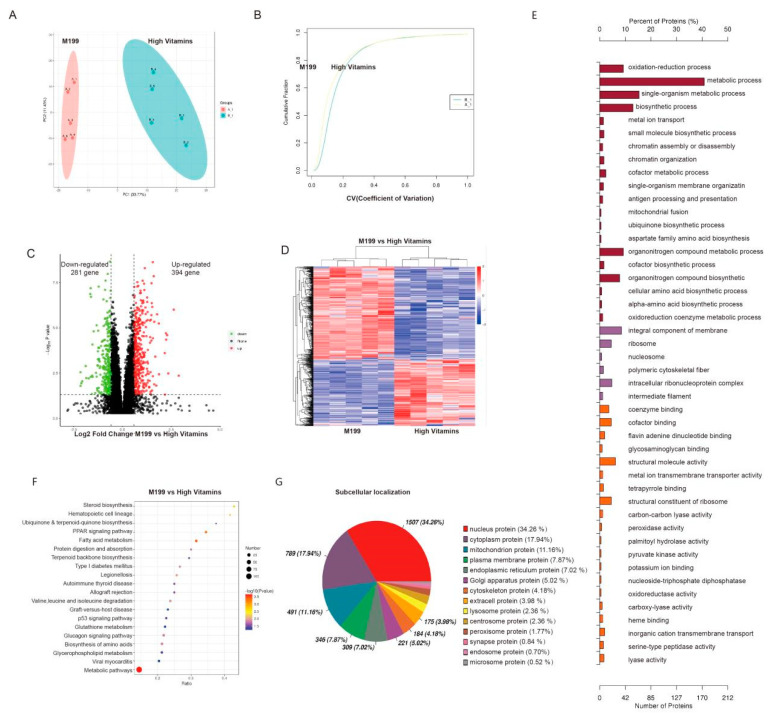
Proteomic analysis. (**A**) PCA analysis. The abscissa PC1 and the ordinate PC2 represent the scores of the principal components ranking, respectively, and the scatter color represents the experimental grouping of samples. (**B**) Repeatability CV analysis. The figure shows the cumulative CV values of all proteins in the corresponding samples, which indicated the repeatability of the sample. (**C**) Differential protein volcano map. The abscissa represents the difference multiple (log2 value) of the differential protein, the vertical axis represents *p*-value (−log10 value), black represents the protein with no-significant difference, red represents the up-regulated and green represents the down-regulated. (**D**) Differential protein-clustering heat map. The vertical is the clustering of samples, and the horizontal is the clustering of proteins. The expression-pattern clustering of protein content among samples can be seen from the vertical clustering. (**E**) Go enrichment histogram shows the enrichment results in three categories, each of which shows up to 20 (*p* value ≤ 0.05). (**F**) KEGG-enrichment bubble diagram. The abscissa in the diagram is the ratio of the number of differential proteins in the corresponding pathway to the number of total proteins identified in the pathway. The size of the dot represents the number of differential proteins in the corresponding pathway. The color of the point represents the *p*-value value of the hypergeometric test. (**G**) Subcellular localization analysis of differential proteins.

To confirm the overall proteins function difference between the two groups, we carried out the principal component analysis (PCA) and coefficient of variation (CV) analysis, which indicated that the degree of variation was small, proving that the repeatability of the samples was good (Figure 3A,B). Both differential protein-clustering volcano map analysis and differential protein-clustering heat map suggested that the addition of vitamins leads to significant changes in protein expression levels in Vero cells (Figure 3C,D).

To determine which biological functions are significantly related to the differential proteins expressed by high-vitamin-culture conditions, we transferred all differential proteins to the gene ontology database. The number of proteins in each term is calculated, and then the hypergeometric test is applied to determine the GO entries that are significantly enriched. The results showed that the most significant biological functions caused by a high-vitamin culture were the oxidation-reduction process, metabolic process, single organization metabolic process, coenzyme binding and cofactor binding, of which the metabolic process enriched the most differential proteins (Figure 3E). Similarly, we performed KEGG pathway enrichment analysis, which showed that high-vitamin culture led to significant enrichment of metabolic-related signal pathways, such as fatty acid metabolism and steroid biosynthesis (Figure 3F). 

Biological cells are a highly ordered structure, which can be divided into different organelles or cell regions according to the spatial distribution and function, such as nucleus, Golgi apparatus, endoplasmic reticulum, mitochondria, cytoplasm and cell membrane. After synthesis in ribosomes, proteins are transported to specific organelles through protein-sorting signals, and some proteins are secreted outside the cell or left in the cytoplasm. Only when proteins are transported to the correct location can they participate in the various life activities of cells. Understanding the subcellular localization information of proteins is very important for the function of organisms. To determine the effect of vitamin addition on protein subcellular localization, the Cell-mPloc 2.0 website was used to analyze the percentage of subcellular localization of different proteins. The results showed that the expression of 131 nuclear proteins and 82 membrane proteins changed significantly (Figure 3G).

### 2.4. Metabolomics Results Surfaced that Vitamin B Affected Unsaturated Fatty Acid Metabolism

Through proteomic detection, we found that vitamin B mainly affects cell proliferation by affecting cell metabolism. To confirm its effect on metabolism, we performed metabolomics detection on 10 cell samples cultured in high-vitamin medium and 10 normal media to confirm the changes in intracellular metabolic levels.

**Figure 4:**
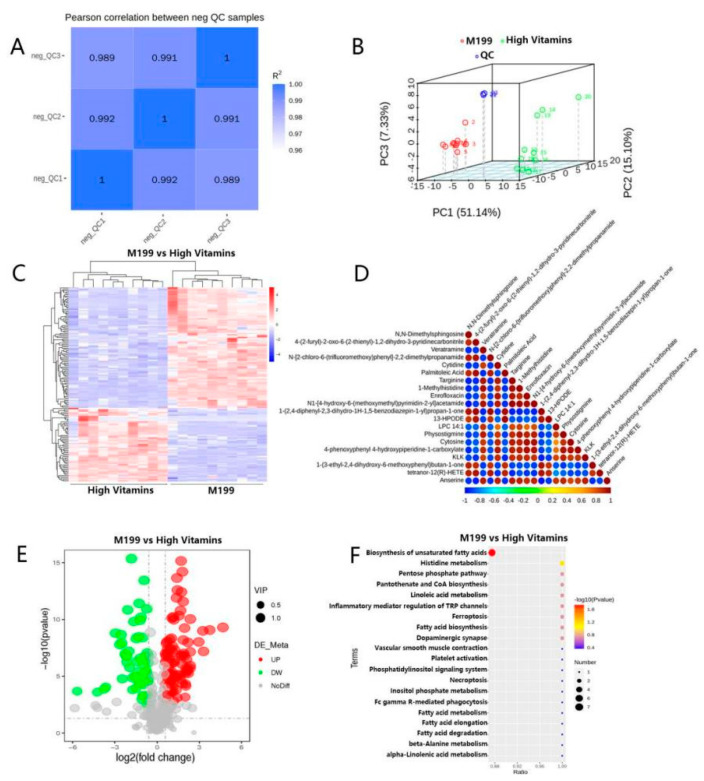
Metabolomics analysis. (**A**) QC sample correlation. The abscissa and ordinate are the QC samples, and the closer the value of R2 is to 1, the better the correlation. (**B**) PCA analysis. The abscissa PC1 and the ordinate PC2 represent the scores of the first and second principal components, respectively, and the scattered points in different colors represent samples from different experimental groups. (**C**) Total differential metabolite-cluster heat map. Hierarchical clustering analysis was performed on the differential metabolites between each comparison pair, and the relative quantitative values of the differential metabolites were normalized, transformed and clustered. The horizontal is the clustering of metabolites, and the vertical is the sample grouping. (**D**) Plot of differential metabolite correlation analysis. By calculating the Pearson correlation coefficient between all the differential metabolites and selecting the top 20 differential metabolites with the significance level *p*-value sorted from small to large for display, you can check the consistency of the metabolite and metabolite change trends. (**E**) Sample comparison versus volcano plot. The volcano plot is drawn according to the VIP value, *p*-value and FC value of the differential metabolites between the comparison pairs, which can visually display the overall distribution of the differential metabolites. (**F**) KEGG-enrichment bubble map: according to the KEGG enrichment results, select the top 20 pathways with *p*-values from small to large to draw a bubble map. The abscissa is x/y (the number of differential metabolites in the corresponding metabolic pathway/the total number of metabolites identified in the pathway); the larger the value, the higher the enrichment of differential metabolites in the pathway. The ordinate is the KEGG pathway name.

Due to the large number of studies, to obtain reliable and high-quality metabolomic data, data quality control should be performed first. The pairwise correlation of QC samples is good (Figure 4A), and the whole detection process is stable. The PCA analysis graph is shown in Figure 4B. To screen the differential metabolites of the two groups of samples, we performed a *t*-test on the relative quantitative values in the samples and calculated the corresponding *p* values. The total differential metabolites-clustering heat map and differential metabolite correlation analysis are shown in Figure 4C,D. Next, we determined up-regulated and down-regulated differential metabolites according to the value of Log2FC, among which, compared with the control group, there were 144 up-regulated metabolites and 194 down-regulated metabolites, among which the differential metabolites with |Log2FC| > 0.5 were total, of which 78 were up-regulated and 132 were down-regulated metabolites (Figure 4E).

Based on the above results, we obtained a variety of significantly different metabolites. To further study the mechanism of action of vitamin supplements and the specific pathways affecting metabolism, we performed functional annotation and pathway analysis of the above differential metabolites. According to the analysis results, the differential metabolites were mainly enriched in the biosynthetic pathways of unsaturated fatty acids (Figure 4F).

## 3. Discussion

Most biological drugs such as vaccines and antibodies rely on the application of mammalian cell matrices, and the production efficiency of cell matrices determines the acquisition rate and quality of target products [6,9]. There are many ways to improve the production efficiency of a cell matrix, but the basic principle is to obtain more cell matrix in the limited, intensive and clean production space in the biopharmaceutical industry to meet the production of target products. A high-efficiency medium is one of the most direct and fundamental ways to improve the production capacity of cell substrates [15,16,17,18]. Existing research work mainly focuses on the correlation analysis combining the cell’s apparent growth state (number, growth rate, etc.) and the optimization of the medium composition, but has not dived into the medium’s response to the cell’s physiological state and the precise metabolic pathway.

In this study, we chose Vero cells as model cells for research because the Vero cell line is one of the most widely used cell matrices in the production of biological products in the world, and the inactivated vaccines produced by the Vero cell line have reached an annual output of 5 billion doses [19,20,21]; the global demand for Vero’s cell-matrix expansion capabilities is enormous. To obtain a high-efficiency and exclusive medium, we systematically screened the nutrients affecting Vero cell proliferation (data unpublished). We found that B vitamins (vitamin B1, vitamin B2 and vitamin B6) can significantly enhance Vero cell proliferation.

Improving the production efficiency of cell substrate mainly starts from the medium and optimizes the medium to promote cell growth by supplementing certain nutrient addition factors. The most commonly used addition factor is newborn calf serum, which can provide the necessary substances for Vero cells to adhere in vitro, but serum is a complex mixture, and the use of serum has many disadvantages and risks [22,23,24,25,26]. Vitamins form a group of essential organic compounds that, in small amounts, perform highly specific functions in cells. It has been documented that vitamin B is essential for the metabolism of carbohydrates, atomic absorption spectroscopy (AAs), and lipids, assisting in enzyme-controlled single- or two-electron redox reactions. Based on this, we adjusted the content of vitamin B and screened and optimized a high-efficiency medium that could achieve high-density cell growth.

To further explain the mechanism of action of the culture medium in colleges and universities, we integrated and analyzed the three omics data of transcriptome, proteome and metabolome [27,28], and quickly screened the data to find the differential proteins and differences that participate in certain metabolic pathways or have the same trend of change. Metabolites provide a data basis for subsequent experimental validation and analysis [29]. Through our analysis, it was confirmed that supplemental vitamin B affects the metabolism-related pathways of Vero cells, affects the high-density growth of Vero cells by affecting the biosynthesis of unsaturated fatty acids and then affects the culture efficiency and yield of the virus [30].

Through this study, we established a high-efficiency exclusive medium for Vero cells, which significantly improved the growth efficiency of cells, providing a new reference for the optimization of the medium and the continuous improvement of productivity of the cell matrix. More importantly, we reversely analyzed the mechanism of the effect of high-efficiency medium on the physiological metabolism of Vero cells; that is, vitamin B promotes cell proliferation by affecting cell metabolism, which is a cell matrix production that cannot be improved by epigenetic optimization. The ability provides important ideas because the revealing of the changing characteristics of this unsaturated fatty acid metabolic pathway points out the direction for us to continuously improve the efficiency of the medium, and then continuously improve the nutrient base and growth environment in the manufacturing process of industrial cell matrix. Thereby, the production capacity of the cell matrix can be continuously improved, and the production efficiency of the target product can be improved. The medium with high vitamin addition can also improve the titer of COVID-19, poliovirus and rabies virus by adjusting the cell density, and reduce the cost of vaccine production. This study provides a new strategy and an important evaluation tool for the continuous improvement of cell-based media in the biopharmaceutical industry.

## 4. Methods

### 4.1. Vero Cells and Culture

Vero cells (No: 88020401) were obtained from the European Collection of Cells (ECACC). Vero cells were cultured in given medium and supplemented with 5% FBS in a humidified incubator with 5% CO_2_ at 37 °C.

M199 medium was commercialized from Gibco (31100035).

High-vitamin medium was self-made by adding vitamin B1 of 0.07 mg/L, B2 of 0.07 mg/L and B6 of 0.175 mg/L to M199 medium.

### 4.2. Cell Counting Kit (CCK)-8 Assay

The effects of high-vitamin B medium on the viability of Vero cell lines were determined by means of a cell counting kit (CCK)-8 assay (Dojindo, Kumamoto, Japan). Vero cells were briefly seeded into 96-well plates at a density of 5 × 10^3^ cells/well with 100 µL of complete medium. After being cultured for 24 h in a humidified incubator with 5% CO_2_ at 37 °C, at indicated time points, 10 μL of CCK-8 solution was added to each well and incubated at 37 °C for 4 h. Optical density (OD) values were measured at 450 nm using a microplate reader (iMark, Bio-Rad, Hercules, CA, USA). Each experiment was performed in triplicate and repeated three times.

### 4.3. Western Blotting

The protein samples separated by PAGE are transferred to a solid-phase carrier (such as nitrocellulose membrane), which adsorbs proteins in a non-covalent form and can keep the types of polypeptides separated by electrophoresis and their biological activities unchanged. The protein or polypeptide on the solid-phase carrier is used as the antigen, and it reacts with the corresponding antibody, and then reacts with the enzyme or isotope-labeled secondary antibody, and the specific purpose of electrophoretic separation is detected by substrate color development or autoradiography.Protein expression was normalized relative to GAPDH expression, the product number was 60004-1-Ig(Proteintech). The KI67 antibody was purchased from Absin; the product number was abs130135. The PCNA antibody product number was 10205-2-AP (Proteintech), and the anti-rabbit secondary antibody product number was PR30011 (Proteintech).

### 4.4. EdU Assay

Cell proliferation was assessed using the cell-light 5-Ethynyl-2′-deoxyuridine (EdU) DNA cell proliferation kit (Ribobio) according to the manufacturer’s instructions: fix Vero cells cultured in M199 medium and Vero cells cultured in high vitamin medium, and fix them with 4% paraformaldehyde at room temperature for 20–30 min; EdU-dye staining was performed after washing with PBS; Hoechst staining was performed after washing with PBS.

### 4.5. RNA Preparation and RT-qPCR

Total RNA was extracted using TRIzol reagent (Invitrogen), and cDNAs were prepared using reverse transcriptase (Thermo Fisher Scientific). Real-time quantitative PCR(RT-qPCR) data were analyzed using the ΔΔCt method, and individual gene expression was normalized to GAPDH RNA expression. GAPDH-F:5′-TGTTGCCATCAATGACCCCT-3′; GAPDH-R: 5′-ATGGAACTTGCCATGGGTGG-3′;

KI67-F: 5′-AAGAAGCAGAGGGTTGCTCC-3′; KI67-R: 5′-TGGGGAGGTCTTCATTTCATTTC-3′;

PCNA-F: 5′-GATATTAGCTCCAGCGGCGT-3′; PCNA-R: 5′-TAGGTGTCGAAGCCCTCAGA-3′.

### 4.6. Transcriptomics Analysis

Fresh cells were washed by PBS and then total RNAs were extracted using the mammalian total RNA extraction kit, following the vendor’s instructions: 5 μL of total RNA solution was taken out to do a concentration and quality measurement. RNA concentration and quality were measured by a Nanodrop 1000 instrument. Total RNAs that had the OD260/OD280 within 1.9–2.2 were considered to be of good quality and were utilized for the further transcriptomic analysis. Each cell line had three biological replicates.

After quantification of total RNA, a multiplexing method was employed for the high-throughput RNA sequencing. Adaptor-ligated cDNA libraries were prepared using the NeoPrep automated microfluidic instrument with the TruSeq Stranded RNA Library Prep Kit (Illumina). PCR-enriched libraries were quantified by Qubit, and equimolar-indexed libraries (different samples had different indexes for multiplexing) were pooled. Pooled libraries were qualitatively checked using the Agilent Tapestation 2200 and quantified using Qubit. The libraries were then diluted to a final concentration of 9 pM and spiked with 2% phiX libraries (Illumina control). Illumina HiSeq. 2500 was used for next-generation transcriptomic sequencing (NGS). The reads generated from different samples could be distinguished based on their different indexes. The quality of the reads was determined by FastQC software. The reads were quality-filtered and mapped on the human genome using NGen software (DNA STAR, Madison, WI). RPKM normalization was then performed prior to the quantification of gene expression by QSeq (DNA STAR). Genes showing > two fold change and *p* < 0.01 were selected as differentially expressed genes and mapped into biological pathways using Ingenuity Pathway Analysis (IPA) software. Differentially expressed gene-cluster annotation and function enrichment were achieved by DAVID. Glycosylation pathways were analyzed by KEGG.

### 4.7. Proteomic Analysis

Three biological replicates of cells from different culture media were suspended in 50 mM ABC buffer (containing 5% SDC) and then homogenized by a beads-beater homogenizer. After lysing, 5 μL of cell lysate was taken out for protein assay using the micro-BCA method (Thermo Scientific/Pierce, Rockford, IL, USA) according to the manufacturer’s instructions. The remaining cell lysate was denatured in an 80 °C water bath for 30 min. Then the denatured proteins were reduced by 5 mM DTT at 60 °C for 45 min, alkylated by 20 mM IAA at 60 °C in darkness for 45 min and followed by quenching the reaction by adding another 5 mM DTT and incubating at 60 °C for 30 min. Next, protease Trypsin-Lys C (Promega, MS gold grade) was added to the sample with a 1:25 enzyme-to-protein ratio and incubated at 37.5 °C for 18 h. After tryptic digestion, formic acid was added (final concentration is 0.5%) to remove the SDC48. After centrifugation at max speed for 10 min, supernatant that contained peptides was collected and dried. Samples were then resuspended with 5% ACN water and ready for proteomic analysis.

Proteomics analysis was performed by LC-MS/MS (Thermo Ultimate 3000 nano system coupled with LTQ Orbitrap Velos) 49. The online purification was performed using a C18 Acclaim PepMap 100 trapping column (75 µm I.D. × 2 cm, 3 µm particle sizes, 100 Å pore sizes, Thermo Scientific, San Jose, CA, USA). Peptide separation was achieved using a C18 Acclaim PepMap RSLC column (75 µm I.D. × 15 cm, 2 µm particle sizes, 100 Å pore sizes, Thermo Scientific, San Jose, CA, USA). A total of 1 μg of proteins was injected for analysis under 60,000 resolution. A 120-min LC method was used for the separation of the digested peptide sample. Briefly, the LC gradient of solvent B in LC-MS/MS analysis was 5% over 10 min, 5–20% over 55 min, 20–30% over 25 min, 30–50% over 20 min, 50–80% over 1 min, 80% over 4 min, 80–5% over 1 min and 5% over 4 min. Two scan events were applied for the data-dependent acquisition. The first scan event was a full MS scan at a resolution of 60 K. The second scan event was a CID MS/MS scan, which fragmented the top 10 intense precursor ions that were selected from the first scan, using the normalized collision energy (CE) of 35%, Q value of 0.25 and 10 ms activation time.

Proteomic data were then converted to a mascot file by Proteome Discover version 1.2 software (Thermo Scientific, San Jose, CA, USA) and then searched against a UniProt database (2014_06, homo sapiens, 20,214 entries) in MASCOT version 2.4 (Matrix Science Inc., Boston, MA, USA). The MASCOT result was verified using Scaffold (version Scaffold_3.6.3, Proteome Software Inc., Portland, OR, USA). Peptide identification probability and protein identification probability were set to 95% and 99%, respectively, and the matched peptide number was 2. A normalized spectra count was applied for quantitation. Then, an additional filter was utilized to remove the proteins that were detected only once in three replicates. Quantitative proteomic datasets from different cell lines were compared and submitted to IPA for pathway analysis and protein-protein interactions.

### 4.8. Metabolomics Analysis

Collect the cell culture medium of each group, centrifuge at 1000× *g* for 10 min, and transfer the supernatant to a new centrifuge tube. This step is mainly used to remove cells: centrifuge at 12,000× *g* for 1 min to remove debris or sediment; transfer the supernatant to a cryopreservation tube and quickly put Quenched in liquid nitrogen for 30 s and temporarily store at −80 °C. All mass spectrometry data were processed for peak picking, alignment and normalization using Progenesis QI data analysis software. Obtain peak intensities for all features. These features were identified by searches in the Metabolite Linkage (METLIN) database or by comparison to standard compounds. Principal component analysis (PCA) and orthogonal partial least squares discriminant analysis were performed on the MS data using the package SIMCA-P.

## Data Availability

I have had access to all the data in the study (for original research articles) and accept responsibility for its validity. I agreed that there is no copyright issue for all the figures in the paper.

## References

[1] Montagnon B.J., Fanget B., Vincent-Falquet J.C. (2010). Industrial-scale production of inactivated poliovirus vaccine prepared by culture of Vero cells on microcarrier. Rev. Infect. Dis..

[2] Montomoli E., Khadang B., Piccirella S., Trombetta C., Mennitto E., Manini I., Stanzani V., Lapini G. (2014). Cell culture-derived influenza vaccines from Vero cells: A new horizon for vaccine production. Expert Rev. Vaccines.

[3] Barrett P.N., Mundt W., Kistner O., Howard M.K. (2009). Vero cell platform in vaccine production: Moving towards cell culture-based viral vaccines. Expert Rev. Vaccines.

[4] Genzel Y., Dietzsch C., Rapp E., Schwarzer J., Reichl U. (2010). MDCK and Vero cells for influenza virus vaccine production: A one-to-one comparison up to lab-scale bioreactor cultivation. Appl. Microbiol. Biotechnol..

[5] Grenier B., Hamza B., Biron G., Xueref C., Viarme F., Roumiantzeff M. (2010). Seroimmunity following vaccination in infants by an inactivated poliovirus vaccine prepared on Vero cells. Rev. Infect. Dis..

[6] Ursache R.V., Thomassen Y.E., van Eikenhorst G., Verheijen P.J.T., Bakker W.A.M. (2015). Mathematical model of adherent Vero cell growth and poliovirus production in animal component free medium. Bioprocess Biosyst. Eng..

[7] Frazatti-Gallina N.M., Mourão-Fuches R.M., Paoli R.L., Silva M.L.N., Miyaki C., Valentini E.J.G., Raw I., Higashi H.G. (2005). Vero-cell rabies vaccine produced using serum-free medium. Vaccine.

[8] Rourou S., van der Ark A., Majoul S., Trabelsi K., van der Velden T., Kallel H. (2009). A novel animal-component-free medium for rabies virus production in Vero cells grown on Cytodex 1 microcarriers in a stirred bioreactor. Appl. Microbiol. Biotechnol..

[9] Nahapetian A.T., Thomas J.N., Thilly W.G. (1986). Optimization of environment for high density Vero cell culture: Effect of dissolved oxygen and nutrient supply on cell growth and changes in metabolites. J. Cell Sci..

[10] Meydani S.N., Ribaya-Mercado J.D., Russell R.M., Sahyoun N., Morrow F.D., Gershoff S.N. (1991). Vitamin B-6 deficiency impairs interleukin 2 production and lymphocyte proliferation in elderly adults. Am. J. Clin. Nutr..

[11] Duthie S.J., Beattie J.H., Gordon M.-J., Pirie L.P., Nicol F., Reid M.D., Duncan G.J., Cantlay L., Horgan G., McNeil C.J. (2014). Nutritional B vitamin deficiency alters the expression of key proteins associated with vascular smooth muscle cell proliferation and migration in the aorta of atherosclerotic apolipoprotein E null mice. Genes Nutr..

[12] Depeint F., Bruce W.R., Shangari N., Mehta R., O’Brien P.J. (2006). Mitochondrial function and toxicity: Role of the B vitamin family on mitochondrial energy metabolism. Chem.-Biol. Interact..

[13] Bieback K., Hecker A., Kocamer A., Lannert H., Schallmoser K., Strunk D., Klüter H. (2009). Human Alternatives to Fetal Bovine Serum for the Expansion of Mesenchymal Stromal Cells from Bone Marrow. Stem Cells.

[14] Füllekrug J., Shevchenko A., Shevchenko A., Simons K. (2006). Identification of glycosylated marker proteins of epithelial polarity in MDCK cells by homology driven proteomics. BMC Biochem..

[15] Shen C.F., Guilbault C., Li X., Elahi S.M., Ansorge S., Kamen A., Gilbert R. (2019). Development of suspension adapted Vero cell culture process technology for production of viral vaccines. Vaccine.

[16] Genzel Y., Rödig J., Rapp E., Reichl U. (2013). Vaccine Production: Upstream Processing with Adherent or Suspension Cell Lines. Anim. Cell Biotechnol..

[17] Akkermans A., Chapsal J.M., Coccia E.M., Depraetere H., Dierick J., Duangkhae P., Goel S., Halder M., Hendriksen C., Levis R. (2020). Animal testing for vaccines. Implementing replacement, reduction and refinement: Challenges and priorities. Biologicals.

[18] Kiesslich S., Kamen A.A. (2020). Vero cell upstream bioprocess development for the production of viral vectors and vaccines. Biotechnol. Adv..

[19] Lazarus J.V., Ratzan S.C., Palayew A., Gostin L.O., Larson H.J., Rabin K., Kimball S., El-Mohandes A. (2020). A global survey of potential acceptance of a COVID-19 vaccine. Nat. Med..

[20] Fiolet T., Kherabi Y., Macdonald C.J., Ghosn J., Peiffer-Smadja N. (2021). Comparing COVID-19 vaccines for their characteristics, efficacy and effectiveness against SARS-CoV-2 and variants of concern: A narrative review. Clin. Microbiol. Infect..

[21] Li M., Wang H., Tian L., Pang Z., Yang Q., Huang T., Fan J., Song L., Tong Y., Fan H. (2022). COVID-19 vaccine development:milestones, lessons and prospects. Signal Transduct. Target. Ther..

[22] Panella S., Muoio F., Jossen V., Harder Y., Eibl-Schindler R., Tallone T. (2021). Chemically Defined Xeno- and Serum-Free Cell Culture Medium to Grow Human Adipose Stem Cells. Cells.

[23] Guinan J., Lopez B.S. (2020). Generating Bovine Monocyte-Derived Dendritic Cells for Experimental and Clinical Applications Using Commercially Available Serum-Free Medium. Front. Immunol..

[24] Badenes S.M., Fernandes T.G., Cordeiro C.S.M., Boucher S., Kuninger D., Vemuri M.C., Diogo M.M., Cabral J.M.S. (2016). Defined Essential 8™ Medium and Vitronectin Efficiently Support Scalable Xeno-Free Expansion of Human Induced Pluripotent Stem Cells in Stirred Microcarrier Culture Systems. PLoS ONE.

[25] Yao T., Asayama Y. (2017). Animal-cell culture media: History, characteristics, and current issues. Reprod. Med. Biol..

[26] Ho Y., Lu H., Ng S. (2021). Applications and analysis of hydrolysates in animal cell culture. Bioresour Bioprocess..

[27] Urrutia M., Blein-Nicolas M., Prigent S., Bernillon S., Deborde C., Balliau T., Maucourt M., Jacob D., Ballias P., Bénard C. (2021). Maize metabolome and proteome responses to controlled cold stress partly mimic early-sowing effects in the field and differ from those of Arabidopsis. Plant Cell Environ..

[28] Schmidt M.A., Barbazuk W.B., Sandford M., May G., Song Z., Zhou W., Nikolau B.J., Herman E.M. (2011). Silencing of soybean seed storage proteins results in a rebalanced protein composition preserving seed protein content without major collateral changes in the metabolome and transcriptome. Plant Physiol..

[29] Kim M., Vogtmann E., Ahlquist D.A., Devens M.E., Kisiel J.B., Taylor W.R., White B.A., Hale V.L., Sung J., Chia N. (2020). Fecal Metabolomic Signatures in Colorectal Adenoma Patients Are Associated with Gut Microbiota and Early Events of Colorectal Cancer Pathogenesis. mBio.

[30] Verdon J., Labanowski J., Sahr T., Ferreira T., Lacombe C., Buchrieser C., Berjeaud J.-M., Héchard Y. (2011). Fatty acid composition modulates sensitivity of Legionella pneumophila to warnericin RK, an antimicrobial peptide. Biochim. Biophys. Acta BBA-Biomembr..

